# Sentinel lymph node mapping using ICG fluorescence and cone beam CT – a feasibility study in a rabbit model of oral cancer

**DOI:** 10.1186/s12880-020-00507-x

**Published:** 2020-09-14

**Authors:** Nidal Muhanna, Harley H. L. Chan, Catriona M. Douglas, Michael J. Daly, Atul Jaidka, Donovan Eu, Jonathan Bernstein, Jason L. Townson, Jonathan C. Irish

**Affiliations:** 1 Department of Otolaryngology - Head & Neck Surgery, University of Toronto, Toronto, ON Canada; 2 Guided Therapeutics (GTx) Program, TECHNA Institute, University Health Network, 101 College St, Toronto, ON M5G 1L7 Canada; 3 Department of Surgical Oncology, Princess Margaret Cancer Centre, Toronto, ON Canada; 4 Department of Otolaryngology, Head and Neck and Maxillofacial Surgery, Tel-Aviv Sourasky Medical Center, Tel Aviv University, Tel Aviv, Israel

**Keywords:** Head and neck surgery, Head and neck cancer, Near-infrared fluorescence imaging, Indocyanine green, Sentinel lymph node biopsy, Cone beam CT

## Abstract

**Background:**

Current sentinel lymph node biopsy (SLNB) techniques, including use of radioisotopes, have disadvantages including the use of a radioactive tracer. Indocyanine green (ICG) based near-infrared (NIR) fluorescence imaging and cone beam CT (CBCT) have advantages for intraoperative use. However, limited literature exists regarding their use in head and neck cancer SLNB.

**Methods:**

This was a prospective, non-randomized study using a rabbit oral cavity VX2 squamous cell carcinoma model (*n* = 10) which develops lymph node metastasis. Pre-operatively, images were acquired by MicroCT. During surgery, CBCT and NIR fluorescence imaging of ICG was used to map and guide the SLNB resection.

**Results:**

Intraoperative use of ICG to guide fluorescence resection resulted in identification of all lymph nodes identified by pre-operative CT. CBCT was useful for near real time intraoperative imaging and 3D reconstruction.

**Conclusions:**

This pre-clinical study further demonstrates the technical feasibility, limitations and advantages of intraoperative NIR-guided ICG imaging for SLN identification as a complementary method during head and neck surgery.

## Background

For patients with early stage (T1/T2) oral squamous cell carcinoma, surgical resection of the primary tumor is typically the preferred first-line treatment. However, a clinical dilemma as to the scope of cervical lymph node management can arise when the patient has a clinically N0 neck, as 20–30% of patients have occult regional disease not detectable on pre-operative imaging or clinical examination [1]. Yet, cervical lymph node metastasis is the single most important predictor of long term survival in oral cavity squamous cell carcinoma, with accurate nodal staging critical for management decisions [2]. In patients with a greater than 20% risk of metastasis, neck dissection is advocated. However, performing an elective neck dissection in all patients who are clinically N0 can result in overtreatment of a proportion of patients with associated morbidity. Another clinical option is to wait and watch patients with a clinically N0 neck, but 8–46% of these patients have occult neck disease and later develop neck metastasis, and many are reluctant to wait when diagnosed with head and neck cancer [1, 3]. As such, techniques that provide additional information to facilitate treatment of the patient at risk for cervical metastases can have significant value.

Sentinel lymph node biopsy (SLNB) is a well-established technique used in many subsites of oncology, including breast cancer, skin cancer and melanoma. In these settings, SLNB has been shown to be sensitive, specific, reduce morbidity and demonstrates survival rates at least as good as with more comprehensive lymph node dissection [4, 5]. The most recent National Comprehensive Cancer Network (NCCN) clinical practice guidelines for head and neck oncology and National Institute for Clinical Excellence (NICE) guidelines addresses the clinical dilemma of the N0 neck in early stage (T1/T2) oral cancer, stating that sentinel lymph node biopsy is an alternative to elective neck dissection for identifying occult cervical metastasis [6]. Despite these guidelines, sentinel lymph node biopsy is not well established or frequently used in clinical practice due to technical difficulties and the tracer commonly used being radioactive.

Indocyanine green (ICG) is a water soluble, tricarbocyanine dye that has Food and Drug Administration (FDA) approval for intravenous injection in many clinical situations [7]. The utility of ICG is based on the properties of functional uptake in the lymph nodes via the lymphatics, radio-opacity on imaging and fluorescence when visualized at surgery. While the scope of literature regarding ICG use for management of head and neck cancer is small in comparison to other anatomic locations, one theme in initial studies is to investigate hybrid approaches in which ICG is combined with other contrast mechanisms such as 99 Tc [8–12]. Here, we investigate a new hybrid approach using ICG fluorescence combined with intraoperative cone-beam CT (CBCT) imaging.

CBCT scanning is increasingly being integrated into interventional procedures as an in-room imaging system. In radiation therapy, recent evidence has demonstrated the value of regular CBCT during treatment delivery to improve the target volume [13]. In interventional radiology, CBCT systems are being used for cerebral angiography and perfusion assessment [14]. Clinical use of CBCT in surgery is in the early stages, but initial work has demonstrated the potential for bony detail and vascular visualization during complex maxillofacial and skull base procedures [15–17]. While ICG alone is currently in clinical use, the lack of studies investigating CBCT imaging for head and neck lymphatics has motived this initial pre-clinical study. Hence, the aim of this study was to evaluate the feasibility of intraoperative CBCT and ICG NIR fluorescence imaging for the identification and resection of sentinel lymph nodes in a rabbit oral cavity squamous cell carcinoma model.

## Methods

**Fig. 1:**
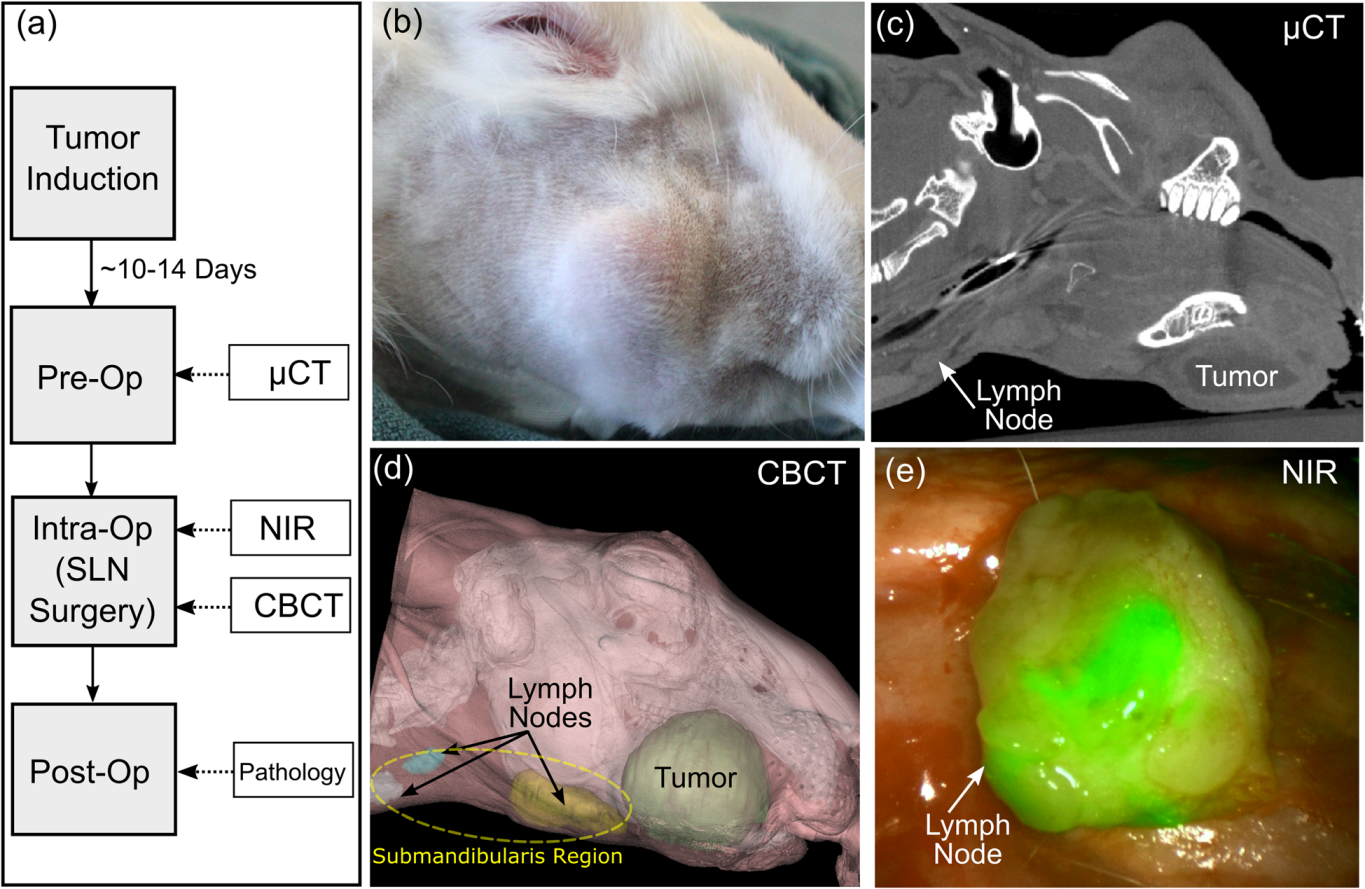
**a** Work flow **b** buccal cancer; **c** microCT demonstrating the tumor and lymph node; **d** surface rendering from microCT. **e** NIR fluorescence view of lymph node

An overview of the study protocol is shown in Fig. 1 and highlights the key steps of tumor induction, pre-operative and intra-operative imaging, and post-operative pathological evaluation. The details of each step are outlined below.

### Animal model

To generate the head and neck tumor model, ten male New Zealand white rabbits (Charles River, Wilmington, Massachusetts) weighing 2.5–3.0 kg were anesthetized with isoflurane and subsequently injected with 300 μL of a high-density cell suspension (5 × 10^6^/mL) of VX2 squamous cell carcinoma into one cheek muscle (buccinator) [18]. Two weeks after VX2 cell injection all ten animals developed tumor in the cheek and cervical lymph node metastases.

### Pre-operative imaging - micro-CT

The development of the primary tumors and lymph node metastases was confirmed and monitored using clinical examination and pre-operative micro-CT imaging (Locus Ultra, GE Healthcare, Milwaukee, Wisconsin). Micro-CT scans were acquired with intravenous (IV) contrast (Omnipaque, GE Healthcare) at 80 kVp and 50 mA. Image reconstruction using traditional filter back projection was performed on a graphic processing unit (GPU) to improve the speed of image reconstruction, resulting in images with 0.15 × 0.15 mm^2^ pixel size and 0.15 mm slice thickness. Cervical lymph nodes were identified on pre-operative imaging. The number of cervical metastasis was recorded for each rabbit.

### Intraoperative imaging - cone-beam CT and near infra-red florescence imaging

CBCT images were acquired intraoperatively with intravenous (IV) contrast (Omnipaque, GE Healthcare) using a prototype flat-panel cone-beam CT system on a mobile C-Arm (Siemens, Erlangen, Germany). Images were acquired at 80 kVp and 2.6 mA using 500 projections over a ~ 178° orbit. Reconstructed volumes had 0.4 × 0.4 mm^2^ pixel size and 0.4 mm slice thickness. Cone beam CT features of the cervical lymph nodes were similar to microCT in that they were enlarged, rounded, cystic, enhanced and hypodense.

**Fig. 2:**
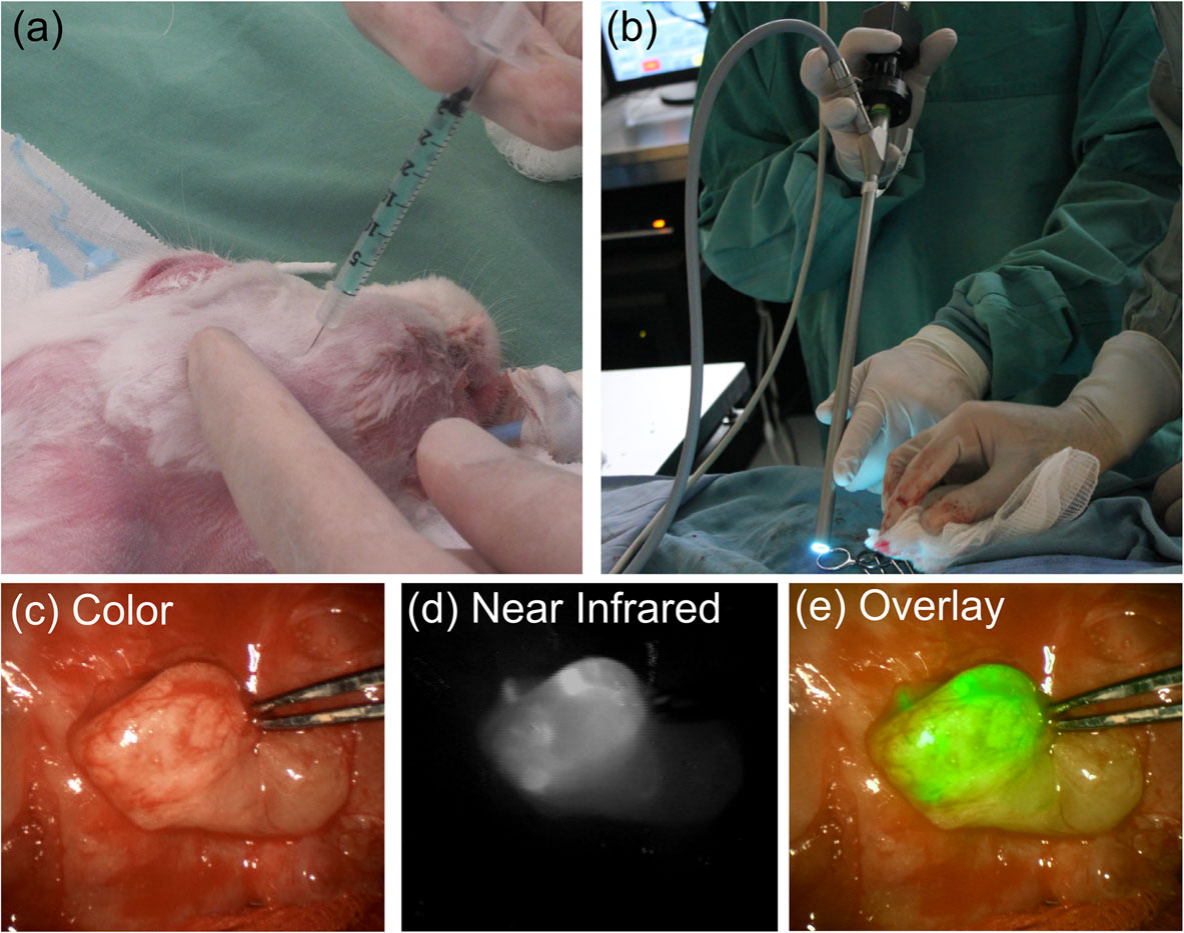
Surgical guidance using intraoperative NIR fluoresence imaging in head and neck surgery with VX2 carcinoma bearing New Zealand White rabbit in buccal area: **a** subcutaneous ICG injection. **b** Intraoperative NIR fluorescence imaging. **c** White light image - lymph node. **d** NIR fluorescence image - lymph node. **e** Fluorescence green pseudo-color image overlay on white light image

Ten to fourteen days after tumor inoculation, rabbits were subcutaneously injected with 50 uL of 0.1 mg/ml ICG solution (IC-GREEN, Akorn, Illinois) at three points around the periphery of the tumor (Fig. 2a). Particular care was taken to minimize ICG leakage from the injection site, to prevent fluorescence contamination in other areas. Thirty minutes post-injection, the shaved neck was imaged for transcutaneous fluorescence using a 0^0^ 10 mm diameter rigid endoscope and the FDA-approved near-infrared device (Endoscopic PINPOINT, Novadaq Technologies Inc., Florida, USA). Color video and the NIR fluorescence images were simultaneously acquired and displayed in real time using the optics equipment and software that separates the color video and NIR fluorescence images and also provides a composite merged image.

Next, a neck skin flap was raised and the subcutaneous tissue was retracted to allow direct visualization of the neck region, to assess the submandibularis nodes, see Fig. 1. The imaging scope distance from the target surface was approximately 7 cm (Fig. 2b). The fluorescence image was used to identify sentinel lymph nodes (Fig. 2c-e). The lymph drainage pattern was observed and imaged and the sentinel node(s) were removed, and sent individually for histopathologic examination.

#### Study end point

All animals were euthanized at the end of the study, in accordance with the protocol approved by the Animal Care Committee of University Health Network, Toronto, Ontario, Canada, with measures to minimize pain and discomfort. Each animal was anesthetized with isofluorane, once the animal was asleep they were then given an intravenous injection of 2.5 mg potassium chloride.

#### Histopathologic evaluation

Tumors and lymph nodes were fixed in formalin after resection, embedded in paraffin blocks, cut and stained with hematoxylin and eosin (H&E) and pan-cytokeratin (AE1/AE3). The slides were scanned and viewed on ImageScope (Leica Microsystems Inc., Illinois, USA).

#### Image analysis

All CT-based image analysis was performed using Microview (GE Healthcare, Milwaukee, WI, USA). Fluorescence intensity was measured using a custom in-house program written using MATLAB (MathWorks®, Natick, Massachusetts).

## Results

### Pre-operative tumor and lymph node detection

**Fig. 3:**
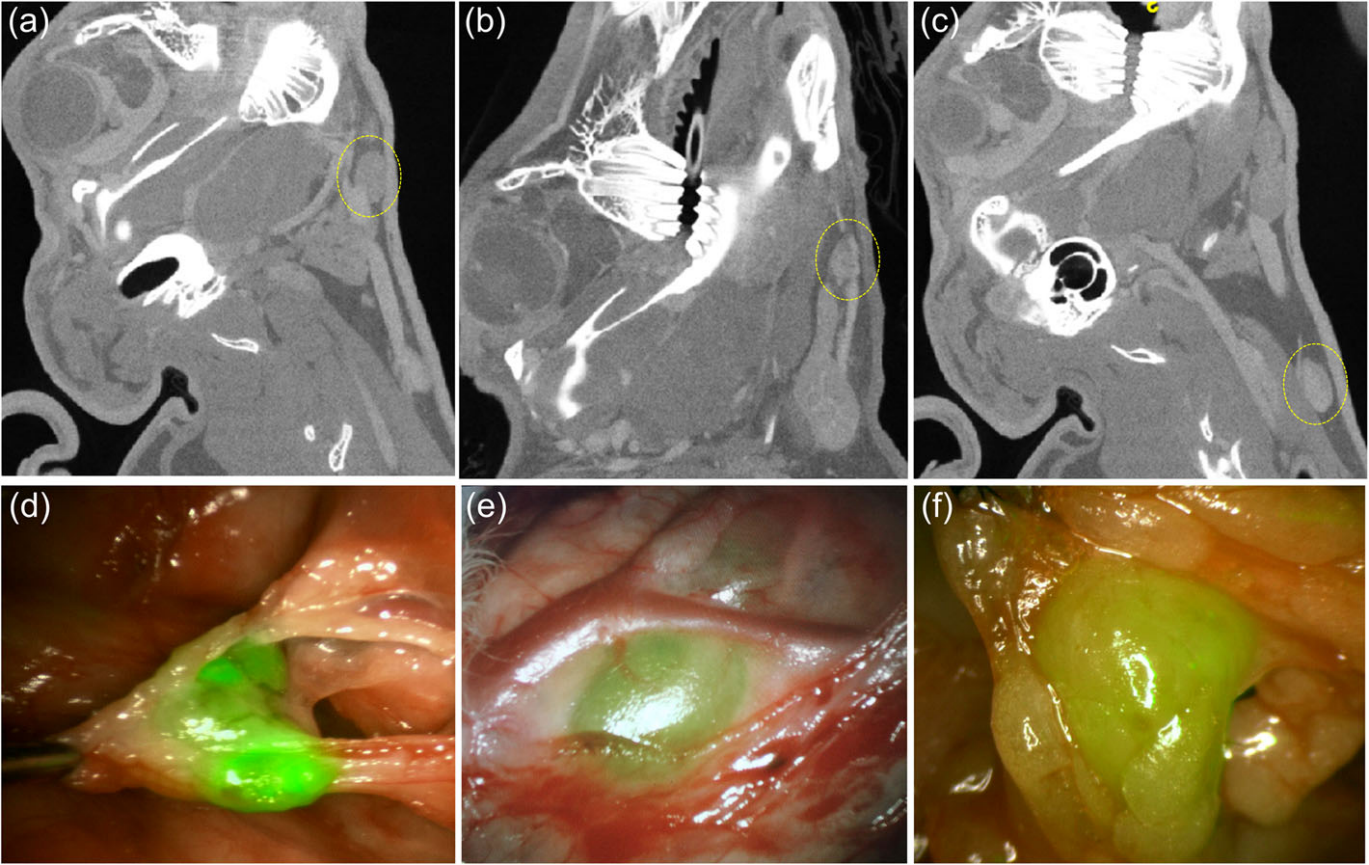
CT [(**a**)-(**c**)] and fluorescence [(**d**)-(**f**)] images of 3 rabbit lymph nodes

Pre-operative microCT imaging was used to monitor tumor and cervical lymph node size as an indicator of metastatic lymph node disease. Images were obtained 10–14 days after tumor induction, when there was clinical evidence of tumor growth and all primary tumors had reached 3 cm in size. Between two and three lymph nodes were detected by digital palpation of the ipsilateral submandibular triangle in all rabbits (Fig. 3), and were considered the SLNs, as confirmed by microCT. A total of 21 SLN were identified on microCT analysis (Table 1). The mean lymph node volume was 1341.4 mm^3^ (SD 83.7). The sensitivity of microCT, when compared to IGC NIR fluorescence was 87.5%, the specificity was 100%.

**Table 1:** Identification of sentinel lymph nodes (SLN) in pre-operative micro CT imaging, intraoperative cone-beam CT (CBCT) imaging, intraoperative fluorescence imaging and final histopathology pathology. Lymph nodes were identified in the submandibularis region, see Fig. 1

Animal	# SLN Pre-op μCT	#SLN Intraoperative CBCT	# SLN Intraoperative Fluorescence	# SLN Metastasis Histopathology
Rabbit 1	3	3	4	1
Rabbit 2	2	2	2	0
Rabbit 3	2	2	2	1
Rabbit 4	2	2	3	1
Rabbit 5	2	2	1	1
Rabbit 6	2	2	3	2
Rabbit 7	2	2	2	2
Rabbit 8	2	2	3	1
Rabbit 9	2	2	2	2
Rabbit 10	2	2	2	1
Total	**21**	**21**	**24**	**12**

### Intraoperative lymph node detection

#### Cone-beam CT

**Fig. 4:**
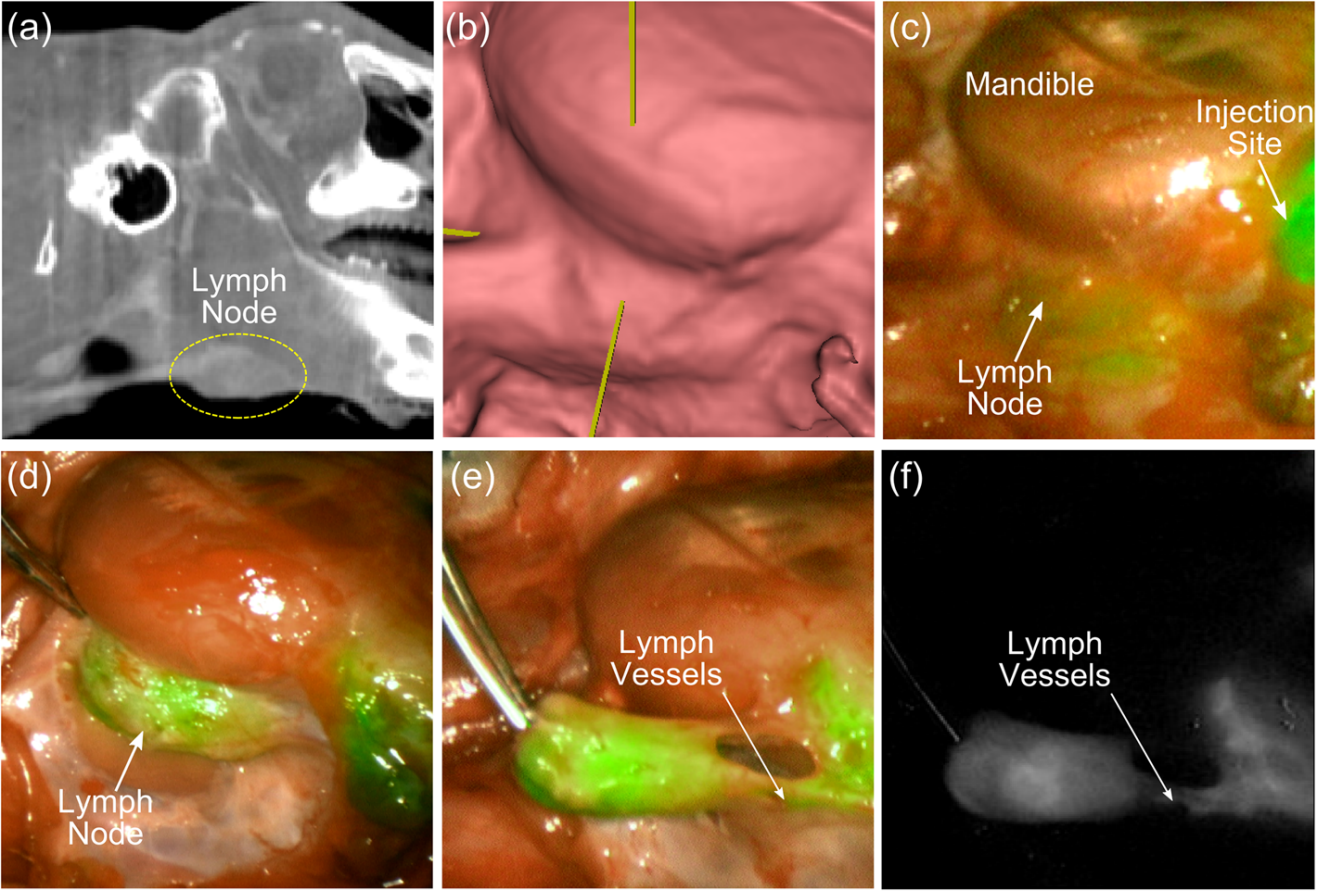
**a** lymph node location on CBCT; **b** Surface rendering from CBCT; **c** lymph node after skin exposure; **d** lymph node after fascia removed; **e** fluorescence in lymph vessels; **f** NIR fluorescence image corresponding to (**e**)

**Fig. 5:**
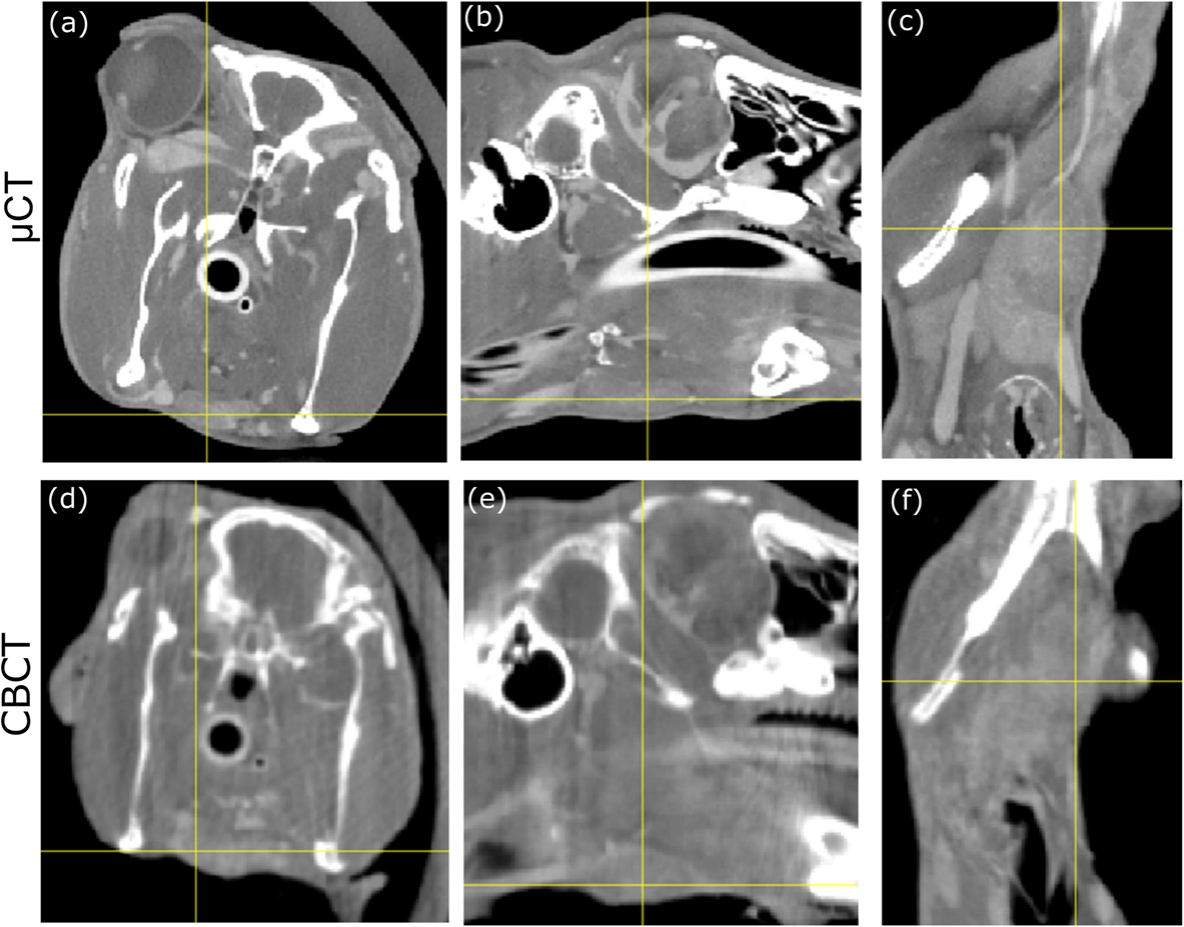
Comparison of pre-operative microCT (top) and intra-operative CBCT images for SLN imaging. **a**,**b**,**c** Axial, coronal and sagittal view on microCT. **d**,**e**,**f** The same rabbit and same views with cone beam CT

Cone-beam CT images were acquired at the time of surgery to intraoperatively identify the anatomical position of the SLNs. As shown in Fig. 4a, bony anatomical landmarks such as the angle of the mandible were easily visible in CBCT images, as well as blood vessel architecture with the use of IV contrast. CBCT images could also be used to generate immediate 3D reconstructions and to assist with landmarking SLNs (Fig. 4b-e). Figure 5 shows a comparison between microCT and CBCT for cervical lymph node visualization; qualitatively, CBCT has inferior soft-tissue resolution due to the blurring from larger voxel edges (0.4 mm for CBCT vs. 0.15 mm for microCT) and well-known cone-beam limitations such as x-ray scatter [19]. Nonetheless, CBCT yielded equivalent performance to microCT for the task of lymph node identification, with a sensitivity of 87.5% and specificity of 100%, as quantified in Table 1.

#### NIR fluorescence

**Fig. 6:**
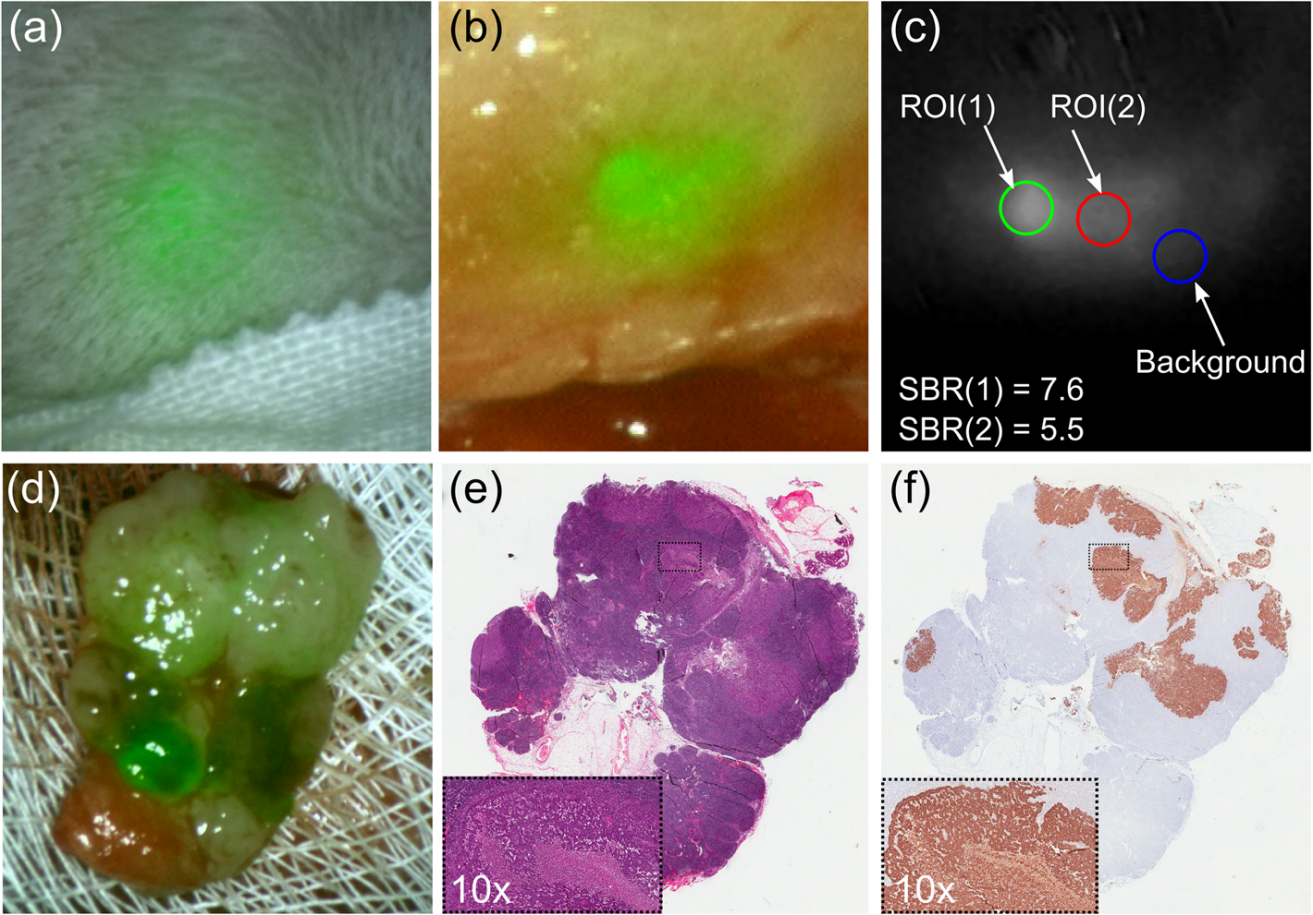
Analysis sequence for lymph node fluorescence imaging in intraoperative surgical guidance resecting the target: **a** percutaneous NIR fluorescence image; **b** in situ NIR fluorescence image; **c** in situ NIR fluorescence image of LN-A in Rabbit 4 with signal-to-background (SBR) ratios, the SBR for region-of-interest 1 and 2 are 6.0 and 4.6, respectively; **d** ex-vivo NIR fluorescence image of resected lymph node; **e** Pathology examination of tissue slice stained with hematoxylin and eosin (H&E); **f** Pathology examination of tissue slice with pan-cytokeratin straining (AE1/AE3). Note: H&E and AE1/AE3 slice suggest the resected lymph node is positive which correspondent to surgeon’s anatomical inspection

Following ICG injection around the periphery of the tumor, NIR fluorescence intraoperative imaging allowed identification of one or more sentinel lymph nodes (range: 1–4 per rabbit) in all ten rabbits. Percutaneous imaging of the SLN was possible prior to raising a neck flap and removal of soft tissues (Fig. 6a). The NIR fluorescence intraoperative identification system was used as an intraoperative tool to map the sentinel nodes to be excised after surgical exposure (Fig. 6b). A total of 24 SLN were identified intraoperatively by NIR fluorescence,no metastasis were missed (Table 1). The average contrast between the fluorescence signal of the SLN and the surrounding tissue, the signal-to-background-ratio (SBR), was 6.68 ± 3.00 (Fig. 6c).

SLN NIR images were taken on average 25–38 min post ICG injection. Histopathological analysis of resected fluorescent positive SLNs, (Fig. 6d), with haematoxylin and eosin staining (Fig. 6e) and pancytokeratin AE1/AE3 (Fig. 6f) showed that 12/24 lymph nodes were positive for metastatic carcinoma.

## Discussion

This study examined the feasibility of using two different intraoperative imaging modalities, cone beam CT and ICG with NIR fluorescence, to help with intraoperative mapping of the sentinel lymph nodes in a rabbit model of head and neck cancer. In addition, pre-operative imaging was performed and compared to the intraoperative NIR Fluorescence SLN yield rate.

### Intraoperative imaging with cone beam CT

Cone beam CT is increasingly being used as an adjunct for improving treatment outcomes in head and neck cancer patients [16]. Recent publications have demonstrated the value of intraoperative cone beam CT scanning in facial trauma surgery, with the CBCT enabling rapid review of cases with revision if necessary [20]. Adaptive radiotherapy has been developed to improve the targeting of radiotherapy; CBCT scanning is done during radiotherapy treatment, with the gross target volume adjusted according to the tumor response of the radiotherapy [21]. The images obtained from the CBCT scanning in this study were of variable quality depending on the tissue being examined. The CBCT scans gave excellent bony resolution, allowing for very quick 3D reconstruction (15 s) and accurate identification of bony landmarks such as the angle of mandible. The use of IV contrast did help with identification of blood vessels within the head and neck region, as we have recently reported on in a clinical study [15]. As expected, the microCT scans had superior soft tissue definition in comparison to CBCT due to well-known limitations of cone-beam imaging [22] Nonetheless, in this study CBCT was able to identify the same number of lymph nodes as microCT. Furthermore, the CBCT system has a distinct advantage in that it has been previously deployed in a clinical OR [16, 17], while microCT is limited to small animal imaging applications. Future work is needed to evaluate CBCT imaging for on-the-table lymph node delineation in patients in comparison to pre-operative diagnostic CT data.

It does, however, highlight the feasibility of using the CBCT in the OR setting for near real-time imaging feedback. Furthermore, we have recently published the value of CBCT in the OR confirming our findings that IV contrast helps with soft tissue delineation.

### Intraoperative imaging with ICG and NIR fluorescence

SLNB in head and neck cancer has not translated into regular clinical practice in many centers, but given that it is now a recommendation in the NCCN guidelines [23] and a quality standard in the NICE guidelines, the technique might be adopted progressively. SLNB is a valuable technique as it enables the identification of the sentinel lymph nodes of the tumor, which can be processed for histology. Only if the SLN is positive for malignancy on histologic examination is a neck dissection subsequently performed, which should reduce operative morbidity in patients who have a negative SLN.

The current techniques used for SLNB include the use of methylene blue dye, radiocolloid and fluorescence guided surgery [8, 9, 24–26]. The current gold standard is the use of preoperative lymphoscintigraphy, intraoperative gamma probe detection with or without the use of blue dye [27]. However, there are a number of disadvantages with this gold standard technique. The preoperative lymphoscintigraphy requires an extra procedure for the patient, often the day before surgery. There are also reports that this stage of the SLNB process is painful for the patient due to the limited use of local anesthetic as it may interfere with results [28]. The use of any radioactive material requires a nuclear physician, with both staff and the patient being exposed to the radiation, although the dose is very low in comparison to the known safe limit. Furthermore, with head and neck cancer specifically, the often close proximity of the primary tumor to the sentinel node can lead to “shine through” from the primary tumor, making it difficult to identify primary tumor from metastasis, particularly in floor of mouth cancers [29].

In every rabbit used in this study, at least 1 SLN was identified, up to a maximum of 4 SLNs. Morton et al. demonstrated, during the MSLT-I trial, that the SLN detection rate in the neck region was only 84.5%, in comparison to 99.3% in the inguinal region and 96.6% in the axillary region [29]. The lower incidence of success in the neck may reflect more complex lymphatic anatomy and smaller lymph nodes. One of the advantages that we have shown with the use of ICG with NIR fluorescence is that imaging for SLN identification can be done quickly after injection of the tumor. We identified the SLNs between 25 and 30 min after injection, which is comparable with van der Vorst et al., who observed that imaging time for identifying SLNs ranged from 5 to 30 min [30]. This shortened time frame is favorable compared to the use of radioactive material which may have to be injected as much as a day before the procedure. The volume of ICG that we injected was small (only 50 uL of 0.1 mg/ml). The dose of ICG injection we used in this study gave a high signal-to-background ratio (SBR) of fluorescence at 6.68 ± 3.00. This again is comparable to the study by van der Vorst et al., who reported a SBR of 8.7 ± 6.4. The small volume of ICG injected with good SBR is reassuring as previous results by Mieog et al. have demonstrated that high doses of ICG can cause quenching, resulting in a decrease in NIR fluorescence signal [31]. A further advantage in using NIR fluorescence in the operating room is the immediate real time feedback obtained when performing the imaging, enabling the surgeon to tailor the procedure using real time imaging feedback. The equipment is small and portable enough that it is not a hindrance to clinical workflow. Toxicity from ICG is rare and primarily due to allergic reaction owing to the small amount of iodine content [32].

### Pre-operative micro CT Vs intraoperative NIR fluorescence imaging

In addition to clinical examination, pre-operative imaging is commonly used to identify enlarged lymph nodes for further biopsy or resection. In the present study, microCT was used in order to preoperatively identify suspicious lymph nodes. ICG identified more lymph nodes than preop micro CT imaging. Our finding that only 12 of 24 enlarged lymph nodes identified by preoperative imaging were histologically positive for metastatic disease fits with findings by Langhans et al. who demonstrated that positive nodes were not larger or different in shape than negative nodes in T1-T2 N0 M0 oral SCC [33].

There are limitations to this study. We did not compare this new approach for identification of SLNs directly with the current gold standard of using radioactive isotope. There were small numbers of animals in the study making firm conclusions difficult to make. Furthermore we do not have longitudinal data to provide information on the dynamic changes in fluorescence of the primary tumour size.

## Conclusion

Overall, this animal model feasibility study demonstrates the practicality of using ICG and intraoperative NIR fluorescence for mapping of sentinel lymph nodes in the VX2 rabbit model. Our study is focused on a combination of CBCT and ICG. Micro CT gave superior soft tissue detail to CBCT, however they had comparable sensitivity in lymph node identification. The combination of CBCT and INR intraoperatively might offer added value to visualize lymph node metastasis in addition to the value received from the Preop CT. This work further demonstrates the potential utility of intraoperative ICG and NIR fluorescence as a method of localizing sentinel lymph nodes in a reliable, safe and cost efficient manner.

## Data Availability

Not applicable.

## Abbreviations

IGS: Image Guided Surgery
STTARR: Spatio-Temporal Targeting and Amplification of Radiation Response
SLNB: Sentinel lymph node biopsy
ICG: Indocyanine green
NIR: Based near-infrared
CBCT: Cone beam CT
NCCN: National Comprehensive Cancer Network
NICE: National Institute for Clinical Excellence
FDA: Food and Drug Administration
IV: Intravenous
H&E: Hematoxylin and eosin
SBR: Signal-to-background-ratio
